# Concordance and Accuracy of Teledermatology Using Mobile Phones in the Outpatient Clinic of Jose R Reyes Memorial Medical Center: Cross-sectional Study

**DOI:** 10.2196/32546

**Published:** 2022-10-31

**Authors:** Ivan Arni Caballero Preclaro, Zharlah Gulmatico-Flores, Elizabeth Amelia Velasco Tianco

**Affiliations:** 1 Department of Dermatology Jose R Reyes Memorial Medical Center Manila Philippines; 2 Department of Dermatology and Venereology Tondo Medical Center Manila Philippines; 3 Department of Dermatology Dr Jose N Rodriguez Memorial Hospital and Sanitarium Caloocan Philippines

**Keywords:** teledermatology, telemedicine, store-and-forward approach, dermatology, virtual consultation, histopathological diagnosis, skin, telehealth, mobile phones, cross-sectional study, dermatologists, dermatologic care, mucosal

## Abstract

**Background:**

Dermatologists rely on visual findings; thus, teledermatology is uniquely compatible to providing dermatologic care. The use of mobile phones in a store-and-forward approach, where gathered data are sent to a distant health provider for later review, may be a potential bridge in seeking dermatologic care.

**Objective:**

This study aimed to determine the agreement between face-to-face consultations and teledermatologic consultations through the store-and-forward approach using mobile phones and its accuracy compared to a histopathologic diagnosis.

**Methods:**

The study design was a cross-sectional study of participants consecutively recruited from dermatology patients who presented with skin or mucosal complaint and without prior dermatologist consultation. Photographs were taken using a standard smartphone (iPhone 6s Plus), and a 4-mm skin punch biopsy was taken on each patient—the gold standard to which the study result was compared to. The photographs were sent to 3 consultant dermatologists using a store-and-forward approach, for independent diagnosis and treatment plan.

**Results:**

A total of 60 patients were included, with a median age of 41 years. There was moderate-to–almost perfect agreement in terms of final diagnosis between the face-to-face dermatologic diagnosis and teledermatologic diagnoses. The third teledermatologist had the highest agreement with the clinical dermatologist in terms of final diagnosis (*κ*=0.84; *P*<.001). Among the 3 dermatologists, there was moderate-to–almost perfect agreement as well. Agreement between pairs of teledermatologists ranged from 0.45 to 0.84. The 3 teledermatologists had moderate-to-substantial agreement with the biopsy results, with the third teledermatologist having the highest accuracy (*κ*=0.77; *P*<.001). Overall, there was a moderate agreement in the diagnosis of patients across raters.

**Conclusions:**

Teledermatology is a viable alternative to face-to-face consultations. Our results show moderate-to-substantial agreement in diagnoses from a face-to-face consultation and store-and-forward teledermatology.

## Introduction

Telemedicine, a subset of eHealth, refers to the use of electronic communications technology for the intention of health management and education [1]. The predominant visual component of the practice of dermatology may translate to a suitable use of telemedicine—hence, the current practice of teledermatology, defined as the use of information and communications technology for the purpose of diagnosis, monitoring, treatment, prevention, research, and education over a distance [2]. This practice is delivered using 2 methods: (1) the store-and-forward method, in which the gathered data are sent to a distant health provider for later review; and (2) the live method, which uses videoconferences to allow consultation in real time between a patient or provider and a distant provider [1].

Telemedicine has been in use since the early 1900s, during which ship captains used the radio to seek medical advice [2]. In modern times, teledermatology has been successfully used with the store-and-forward method, such as in the Africa Teledermatology Project, the Swinfen Charitable Trust, the Médecins Sans Frontières Telemedicine Network, and the Réseau Afrique Francophone de Télémédecine project [1]. The African Teledermatology Project connected sub-Saharan countries to dermatologists from resource-rich countries to provide dermatologic care [3]. In Mongolia, Byamba et al [4] assessed the costs and efficiency of teledermatology against face-to-face consultations. It lessened the costs and time of travel, decreased the time to seek dermatologic care, and improved patient satisfaction.

Applications of teledermatology includes teletriage, primary care–to-dermatology consultation, specialists-to-dermatology consultation, dermatologist-to-dermatologist consultation, telepathology, long-term management, care coordination, and dermatology education [1]. The success of such applications was found to be due to satisfactory skin diagnosis and disease management, its diagnostic concordance with face-to-face visits, and the satisfaction of both patient and health provider with the format [3-11].

With only 1063 board-certified dermatologists in the Philippines, the ratio of dermatologists to the total population is still low. There is limited distribution of dermatologists to rural areas. With skin diseases as one of leading causes of disability worldwide, traditional methods of consultation have been a challenge; thus, there is a need for innovative methods and platforms to provide adequate care over a great distance. In recent advances in teledermatology, several studies have dealt with the use of mobile devices such as smartphones as a tool to convey clinical information [3,5-12]. Out of a total population of 100 million Filipino people, 70 million own a mobile phone [5]. Mobile phones may serve as a bridge to other areas lacking dermatologic care, providing a solution to the challenges of the lack of health provider and distance.

In a resource-limited country, specialist care is not readily available to many patients. There is a great disproportion of specialists to the overall population. Compared to resource-rich countries, there is less effort to promote the use of telemedicine due to a smaller return of investment and lack of technical infrastructures necessary to provide care for our patients [6].

Teledermatology should be implemented in a way that is sensitive to the culture and unique needs of the local setting, bearing in mind limitations of resources. Teledermatology comes with its own challenges such as sustainability in terms of setting up the platform, the computer literacy of patients and health care providers, the regularity and availability of internet access and mobile network connectivity, the sensitivity of patients wherein their preference is face-to-face contact or they have resistance to being photographed, patient privacy and data security, as well as the setup for payment [7].

Teledermatology is deemed to be the future of the practice of dermatology as evidenced by the number of available dermatologists and their practices being commonly clustered around urban localities [7]. Its practice is even more relevant due to the COVID-19 pandemic, wherein physical distancing is one of the key components of transmission prevention. The use of eHealth through teledermatology can ease the anxiety experienced by patients when faced with the possibility of needing a face-to-face consultation as well as stemming the overwhelming need for specialty consultations in remote rural municipalities. Teledermatology can thus provide a means of getting consultations while maintaining public health safety. Beyond practicing amid a pandemic, teledermatology may increase the access of the population to specialists who are physically too far away. This study aimed to determine the agreement and the accuracy of face-to-face consultations and teledermatologic consultations with the store-and-forward approach using a mobile phone. Additionally, we aimed to determine interrater concordance (ie, statistical agreement) between the clinical face-to-face dermatologist and teledermatologists in diagnosis, the interrater concordance in diagnoses among the teledermatologists, and the accuracy of teledermatologic diagnoses with the histopathology diagnosis.

## Methods

### Study Design and Setting

This was a cross-sectional study conducted at the outpatient department (OPD) of the Jose R. Reyes Memorial Medical Center from August 1 to September 30, 2018. Face-to-face consultations were done at the dermatology clinic of the OPD, whereas teledermatology diagnoses were performed independently by 1 or 2 dermatologists.

### Ethics Approval

Prior to implementation, the study was approved by the hospital institutional review board (protocol number 18-015) and adhered with the ethical standards of the committee on human experimentation with the Helsinki Declaration of 1975.

### Participants

The primary investigator consecutively recruited dermatology patients—Filipino patients of any age and sex who presented with any skin or mucosal complaint during their first consultation for that specific complaint. Patients who came in for a follow-up check-up, had previously been biopsied for the same skin lesion, who came in with a diagnosis already previously known to the patient, or had previously been evaluated by a dermatologist for the same skin or mucosal lesion were excluded from the study.

### Data Collection

All patients received a face-to-face clinical evaluation by a supervising clinical dermatologist (CD) that was assisted by the primary investigator according to the standard procedure at the OPD. After evaluation, the patients were invited to participate in the study. Written informed consent was obtained from adults and parents of pediatric patients. If the patient, or legal guardian for a minor patient, consented to participate in the study, the primary investigator then proceeded to conduct a protocol-based dermatologic evaluation for this study. The skin or mucosal lesions were photographed using an iPhone 6s Plus with a 12-megapixel back camera. Photographs were taken 4 inches (10 cm) away, perpendicular to the lesion under ambient lighting. The primary investigator obtained a 4-mm skin punch biopsy on the skin or mucosal lesion of interest. The patients were prescribed treatment based on the clinical diagnosis made from this face-to-face clinical evaluation.

### Diagnosis From Teledermatology

The photographs from the iPhone 6s Plus were viewed separately by 3 teledermatologists. They were provided with the patient’s age and sex, a brief description of the patient’s medical history, and high-resolution images of the skin lesion(s). The teledermatologists gave their clinical diagnosis and proposed a treatment plan for each patient.

### Statistical Analysis

A minimum of 56 study participants were required for this study, assuming an 18% probability of disagreement between the CD and teledermatologist, a 95% CI of plus or minus 0.10, and 5% level of significance, based on Lamel et al [8] and Machin et al [9].

Descriptive statistics were used to summarize the general and clinical characteristics of the participants. Frequency and proportion were used for nominal variables, median and range for ordinal variables, and mean and SD for interval or ratio variables. Cohen *κ* was used to determine statistical agreement between the diagnoses of the CD and teledermatologists. All valid data were included in the analysis. Missing variables were neither replaced nor estimated. Null hypothesis was rejected at .05 α-level of significance. Stata statistical software (version 15.0; StataCorp) was used for data analysis.

## Results

### Patient Demographics and Disease Categories

A total of 60 patients were included in the study, with a median age of 41 (range 4 months to 75 years) years, and 50% (n=30) were female (Table 1).

**Figure 1:**
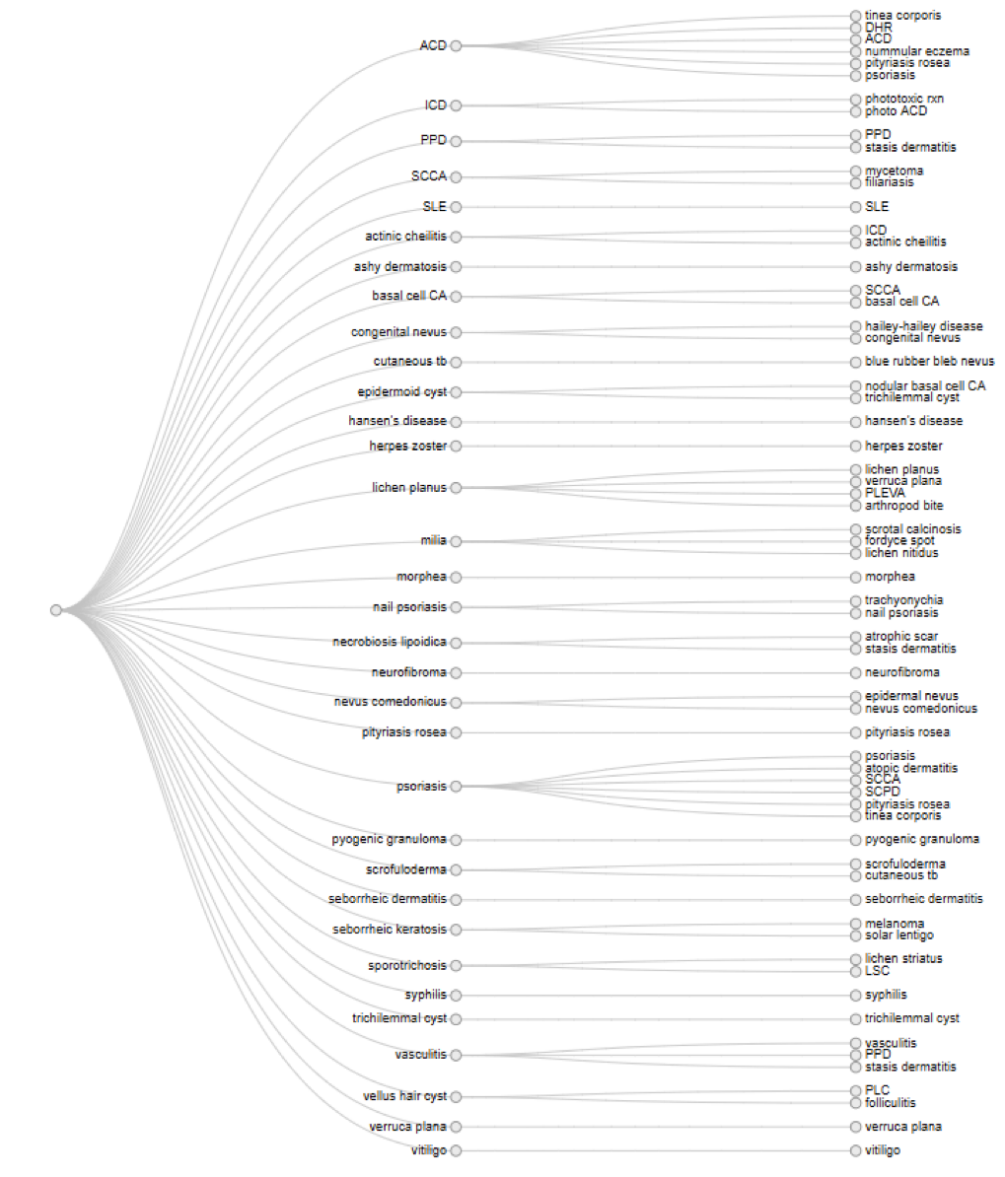
Cluster dendogram comparing face-to-face dermatologic diagnoses versus teledermatologic diagnoses and differentials. ACD: allergic contact dermatitis; CA: carcinoma; DHR: dermal hypersensitivity reaction; ICD: irritant contact dermatitis; LSC: lichen simplex chronicus; PLEVA: pityriasis lichenoids et varioliformis acuta; PLC: pityriasis lichenoides chronica; PPD: pigmented purpuric dermatosis; SCCA: squamous cell carcinoma; SCPD: subcorneal pustular dermatosis; SLE: systemic lupus erythematosus.

There were 57 dermatologic diagnoses identified from both the CD and 3 teledermatologists. The 3 teledermatologists were board-certified dermatologists who have been practicing for 3 to 7 years. The diagnoses from face-to-face dermatology and teledermatology are enumerated on Figure 1.

The diagnoses confirmed by histopathology were classified by standard disease categories (Table 2). A majority (n=31, 52%) of the diseases fell under the inflammatory disease category, followed by benign neoplasms (n=11, 18%). Other disease categories include infectious diseases, vascular diseases, and malignant neoplasms.

**Table 1:** Demographic and clinical profile of patients (N=60).

Characteristic		Value
Age (years), median (range)		41 (0.33-75)
**Sex, n (%)**		
	Male	30 (50)
	Female	30 (50)
**Comorbidities, n (%)**		
	Hypertension	3 (5)
	Benign prostate hypertrophy	2 (3)
	Diabetes	2 (3)
	Dyslipidemia	2 (3)
	Allergy	1 (2)
	Heart disease	1 (2)

**Table 2:** Disease categories based on biopsy (N=60).

Dermatologic disease category	Diagnosis, n (%)
Inflammatory	31 (52)
Benign neoplasm	11 (18)
Infectious	8 (13)
Vascular	6 (10)
Malignant neoplasm	4 (7)

### Face-to-Face Dermatologic Diagnosis Versus Teledermatologists’ Diagnoses

The concordance rates between the CD and teledermatologists were from 57.1% to 86.7%. There was moderate-to–almost perfect agreement in terms of final diagnosis between the face-to-face dermatologic diagnosis and teledermatologic diagnoses (Table 3). Teledermatologist 3 had almost perfect agreement with the clinical dermatologist in terms of final diagnosis (*κ*=0.84; *P*<.001).

**Table 3:** Agreement between clinical dermatologist and teledermatologists based on final diagnosis (N=60).

Agreement	Concordance (%)	*κ* value^a^	Interpretation	*P* value
CD^b^ vs T1^c^	57.1	0.55	Moderate agreement	<.001
CD vs T2	60.4	0.58	Moderate agreement	<.001
CD vs T3	86.7	0.84	Almost perfect agreement	<.001

^a^*κ* interpretation: ≤0, poor; 0-0.2, slight; 0.21-0.40, fair; 0.41-0.60, moderate; 0.61-0.80, substantial; 0.81-1.00, almost perfect.

^b^CD: clinical dermatologist.

^c^T: teledermatologist.

### Agreement Across Teledermatologists

The concordance rates among the teledermatologists were from 46.8% to 86.7%. Among the 3 dermatologists, there was moderate-to–almost perfect agreement as well (Table 4). Agreement between pairs of teledermatologists ranged from 0.45 to 0.84. Teledermatologists 1 and 3 had an almost perfect agreement (*κ*=0.84; *P*<.001).

**Table 4:** Agreement among teledermatologists based on final diagnosis (N=60).

Agreement	Concordance (%)	*κ* value^a^	Interpretation	**P* value*
T1^b^ vs T2	46.8	0.45	Moderate agreement	<.001
T1 vs T3	86.7	0.84	Almost perfect agreement	<.001
T2 vs T3	73.3	0.69	Substantial agreement	<.001

^a^*κ* interpretation: ≤0, poor; 0-0.2, slight; 0.21-0.40, fair; 0.41-0.60, moderate; 0.61-0.80, substantial; 0.81-1.00, almost perfect

^b^T: teledermatologist.

### Teledermatologists Versus Histopathology

The accuracy rates of the teledermatologists were from 60% to 80%. The 3 teledermatologists had moderate-to-substantial agreement with the biopsy results (Table 5). Teledermatologist 3 had the highest accuracy in diagnosing diseases (*κ*=0.77; *P*<.001).

**Table 5:** Agreement between teledermatologists and biopsy based on final diagnosis (N=60).

Agreement	Concordance (%)	*κ* value^a^	Interpretation	*P* value
Biopsy vs T1^b^	60	0.58	Moderate agreement	<.001
Biopsy vs T2	62.8	0.61	Substantial agreement	<.001
Biopsy vs T3	80	0.77	Substantial agreement	<.001

^a^*κ* interpretation: ≤0, poor; 0-0.2, slight; 0.21-0.40, fair; 0.41-0.60, moderate; 0.61-0.80, substantial; 0.81-1.00, almost perfect

^b^T: teledermatologist.

### Overall Agreement

The *κ* values in the present study were from 0.53 to 0.58. The agreement between the teledermatologists and biopsy was the highest. However, there was still a moderate agreement in the diagnosis of patients among raters, based on final diagnosis (Table 6). The overall agreement per specific diagnosis is shown in Multimedia Appendix 1.

Based on the disease categories, the CD and teledermatologists had moderate-to-substantial agreement (Table 7). Vascular diseases, inflammatory diseases, and benign neoplasm showed substantial agreement with *κ* values from 0.64 to 0.72. Conversely, malignant neoplasm and infectious diseases showed moderate agreement with *κ* values from 0.58 to 0.60.

**Table 6:** Summary of overall agreement among raters based on final diagnosis. The number of ratings per subject vary; thus, we could not calculate test statistics (*P* value).

Agreement	*κ* value^a^	Interpretation
Clinical dermatologist and teledermatologists	0.56	Moderate agreement
Among teledermatologists	0.53	Moderate agreement
Teledermatologists and biopsy	0.58	Moderate agreement

^a^*κ* interpretation: ≤0, poor; 0-0.2, slight; 0.21-0.40, fair; 0.41-0.60, moderate; 0.61-0.80, substantial; 0.81-1.00, almost perfect.

**Table 7:** Agreement of all raters based on disease category. The number of ratings per subject vary; thus, we could not calculate test statistics (*P* value).

Disease category	*κ* value^a^	Interpretation
Inflammatory	0.64	Substantial agreement
Infectious	0.58	Moderate agreement
Benign neoplasm	0.62	Substantial agreement
Malignant neoplasm	0.60	Moderate agreement
Vascular	0.72	Substantial agreement

^a^*κ* interpretation: ≤0, poor; 0-0.2, slight; 0.21-0.40, fair; 0.41-0.60, moderate; 0.61-0.80, substantial; 0.81-1.00, almost perfect.

## Discussion

This study aimed to find the agreement and accuracy of face-to-face consultations and teledermatologic consultations with the store-and-forward approach. Overall, there was a moderate agreement in the diagnosis of patients among raters. The concordance rates of teledermatologists with that of face-to-face dermatologist and the accuracy of teledermatologists with the biopsy results were consistent with the previous studies that used mobile phone teledermatology. The accuracy of mobile phone dermatology was low compared to other media in teledermatology [10-12].

Similar results can be found in other studies. For instance, Clark et al [10] reviewed 15 studies that used mobile phones in teledermatology. Concordance is the reliability or agreement between the face-to-face dermatologist and teledermatologist. The diagnostic concordance rates of teledermatology using mobile phones ranged from 40% to 95%, whereas the management concordance rates ranged from 69% to 100%. Varying results have been documented for both diagnostic and management concordance rates in 41% to 94% of cases [11,12]. Despite having high concordance rates, the study concluded that traditional face-to-face dermatology is still superior to mobile phone teledermatology. In this study, both concordance rates showed moderate-to-substantial agreement. Therefore, the results in this study are consistent with the results of the systematic review.

Although the results show that there is a moderate overall agreement in diagnosis, other factors that make up the process must be studied to determine how ready an institution or country is for teleconsultations. However, these results may be useful for exploring the possibility of teleconsultations in other fields. The results may also be used as a reference for learning more about the common practices used in telemedicine that are unique to the community’s culture, norms, and needs. Future studies that develop the subject may look into these areas and may also test other populations’ readiness. It is recommended to look into other demographic factors that may explain the results, such as the technological access and literacy of patients and health care providers involved in the treatment process.

Other technical factors can affect accurate diagnoses in skin diseases, including, but not limited to, image resolution and image quality (particularly color balance and brightness). Image resolution pertains to the number of pixels in a picture [13]. For this study, the phone used (iPhone 6s Plus) has a camera that generates an image with a 12-megapixel resolution, which entails a pixel resolution of approximately 4290 × 2800 pixels, with 4K HD recording resolution capability of 3840 × 2160 [14]. The American Telemedicine Association requires a minimum of 640 × 360 resolution for pictures and 30 frames per second for videos to see a patient via telemedicine, which makes the smartphone qualified to be used for teledermatology purpose [15]. Image quality, meanwhile, is defined as the accuracy of the image’s representation of details stored in pixels [13]. Brightness is the intensity of light reflected from objects, captured by a camera; color balance is the “color temperature” or the relative warmth or coolness of white light in a picture [16]. It was pointed out by Iyatomi et al [17] that accurate color information is important for melanoma diagnoses, and incorrect brightness and color balance adversely impact diagnostic performance. They were able to develop a color calibration filter that automatically adjusts the image quality of a melanoma to help diagnosticians correctly identify melanoma types. The principle of correctly calibrated images can also be applied to other skin diseases. Friedman et al [18] asked 13 dermatologists to anonymously review 13 clinical images of a fungal skin infection and found that the majority of the cases were identified correctly 50% of the time, with only 1 of the cases identified correctly 90% of the time.

Advances in artificial intelligence enables more accurate and faster diagnoses of skin diseases, interfacing with teledermatology. A deep learning system developed by Liu et al [19,20] was able to distinguish 26 common skin diseases, with results considered as noninferior to 3 board-certified dermatologists and superior to primary care physicians and nurse practitioners involved in the study. The data consist of 17,777 deidentified cases collected from a teledermatology service. Another deep learning model developed by Esteva et al [21] was trained with a data set of 129,450 images, consisting of 2032 diseases. Its performance was tested against 21 board-tested dermatologists to perform 2 tasks, which are classifying images correctly as: (1) having keratinocyte carcinomas versus benign seborrheic keratoses, and (2) having malignant melanomas versus benign nevi. The model was able to match the performance of the experts, further showing that artificial intelligence can be leveraged to critically deliver appropriate diagnostic care. Due to the successful compression and optimization achieved by these neural networks, interfacing using apps installed on mobile phones or websites is entirely possible, making access to these tools easier.

It must be noted that this study was conducted prior to the COVID-19 pandemic. Hence, it was in a setting where teledermatology was a “proof of concept” for diagnosis based on phone images, rather than done in a real-life setting, and for which the diagnosticians had no binding physician-patient relationship. It is likely that the practice of teledermatology in more recent times may even perform better now that it is rapidly becoming culturally acceptable in clinical practice. In conclusion, teledermatology is a viable alternative to face-to-face consultations. This study showed moderate-to-substantial agreement in diagnoses from face-to-face consultation and store-and-forward teledermatology.

## Appendix

Multimedia Appendix 1 Agreement between teledermatologists and biopsy results.

## Abbreviations

CD: clinical dermatologist
OPD: outpatient department

## References

[1] Williams V, Kovarik C (2018). Long-range diagnosis of and support for skin conditions in field settings. Trop Med Infect Dis.

[2] Coates SJ, Kvedar J, Granstein RD (2015). Teledermatology: from historical perspective to emerging techniques of the modern era: part I: history, rationale, and current practice. J Am Acad Dermatol.

[3] Weinberg J, Kaddu Steven, Gabler Gerald, Kovarik Carrie (2009). The African Teledermatology Project: providing access to dermatologic care and education in sub-Saharan Africa. Pan Afr Med J.

[4] Byamba K, Syed-Abdul S, García-Romero M, Huang CW, Nergyi S, Nyamdorj A, Nguyen PA, Iqbal U, Paik K, Celi L, Nikore V, Somai M, Jian WS, Li YC (2015). Mobile teledermatology for a prompter and more efficient dermatological care in rural Mongolia. Br J Dermatol.

[5] Abadilla EV (2016). Smartphone users up 25% to 32.5 M. Manila Bulletin.

[6] Combi C, Pozzani G, Pozzi G (2016). Telemedicine for developing countries. a survey and some design issues. Appl Clin Inform.

[7] Pasquali P, Sonthalia Sidharth, Moreno-Ramirez David, Sharma Pooram, Agrawal Mahima, Gupta Somesh, Kumar Dinesh, Arora Dharmendra (2020). Teledermatology and its current perspective. Indian Dermatol Online J.

[8] Lamel SA, Haldeman KM, Ely H, Kovarik CL, Pak H, Armstrong AW (2012). Application of mobile teledermatology for skin cancer screening. J Am Acad Dermatol.

[9] Machin D, Campbell MJ, Tan SB, Tan SH (2018). Sample Sizes for Clinical, Laboratory and Epidemiology Studies. 4th ed.

[10] Clark AK, Bosanac S, Ho B, Sivamani RK (2018). Systematic review of mobile phone-based teledermatology. Arch Dermatol Res.

[11] Whited JD, Hall R P, Simel D L, Foy M E, Stechuchak K M, Drugge R J, Grichnik J M, Myers S A, Horner R D (1999). Reliability and accuracy of dermatologists' clinic-based and digital image consultations. J Am Acad Dermatol.

[12] Kvedar JC, Edwards R A, Menn E R, Mofid M, Gonzalez E, Dover J, Parrish J A (1997). The substitution of digital images for dermatologic physical examination. Arch Dermatol.

[13] Tabora V (2019). The relationship of image quality and image resolution. Medium.

[14] Giordano R (2005). Megapixels chart. Design215.

[15] Hume R, Looney J (2016). Designing for telemedicine spaces. ASHE Health Facilities Management.

[16] (2020). Tutorials: white balance. Cambridge in Color.

[17] Iyatomi H, Celebi M Emre, Schaefer Gerald, Tanaka Masaru (2011). Automated color calibration method for dermoscopy images. Comput Med Imaging Graph.

[18] Friedman A, George Washington University (2016). Fungal skin infections commonly misdiagnosed. Science Daily.

[19] Liu Y, Bui P (2019). Using deep learning to inform differential diagnoses of skin diseases. Google AI Blog.

[20] Liu Y, Jain Ayush, Eng Clara, Way David H, Lee Kang, Bui Peggy, Kanada Kimberly, de Oliveira Marinho Guilherme, Gallegos Jessica, Gabriele Sara, Gupta Vishakha, Singh Nalini, Natarajan Vivek, Hofmann-Wellenhof Rainer, Corrado Greg S, Peng Lily H, Webster Dale R, Ai Dennis, Huang Susan J, Liu Yun, Dunn R Carter, Coz David (2020). A deep learning system for differential diagnosis of skin diseases. Nat Med.

[21] Esteva A, Kuprel B, Novoa Ra, Ko J, Swetter Sm, Blau Hm, Thrun S (2017). Dermatologist-level classification of skin cancer with deep neural networks. Nature.

