# Supplementary material for: Concordance and Accuracy of Teledermatology Using Mobile Phones in the Outpatient Clinic of Jose R Reyes Memorial Medical Center: Cross-sectional Study
Source: JMIR Dermatol. 2022 Oct 31;5(4):e32546. doi: 10.2196/32546 (PMC10334942; doi:10.2196/32546)
Supplement: Multimedia Appendix 1 [file derma_v5i4e32546_app1.docx]

Multimedia Appendix 1

The following diseases had a high agreement between teledermatologists and biopsy results: Ashy dermatosis, Herpes zoster, Intradermal nevus, Neurofibroma, Pemphigus vulgaris, Pyogenic granuloma, Seborrheic dermatitis, Syphilis, Vasculitis, Vitiligo, Morphea, and Systemic lupus erythematosus.

**Multimedia Appendix 1. Agreement between teledermatologists and biopsy results.**

| **Disease** | **Kappa** |
| --- | --- |
| Ashy dermatosis | 1 |
| Herpes zoster | 1 |
| Intradermal nevus | 1 |
| Neurofibroma | 1 |
| Pemphigus vulgaris | 1 |
| Pyogenic granuloma | 1 |
| Seborrheic dermatitis | 1 |
| Syphilis | 1 |
| Vasculitis | 1 |
| Vitiligo | 1 |
| Morphea | 1 |
| Systemic lupus erythematosus | 1 |
| Pityriasis rosea | 0.86 |
| Psoriasis | 0.82 |
| Verruca plana | 0.78 |
| Allergic contact dermatitis | 0.5 |
| Basal cell carcinoma | 0.49 |
| Arthropod bite | 0.46 |
| Atrophic scar | 0.46 |
| Nevus comedonicus | 0.46 |
| Solar lentigo | 0.46 |
| Hansen's disease | 0.46 |
| Scrofuloderma | 0.46 |
| Drug eruption | 0.45 |
| Pigmented purpuric dermatosis | 0.45 |
| Trichilemmal cyst | 0.38 |
| Inflammatory linear verrucous epidermal nevus | 0.27 |
| Squamous cell carcinoma | 0.23 |
| Fordyce spot | 0.19 |
| Trachyonychia | 0.19 |
| Pityriasis lichenoides chronica | 0.19 |
| Folliculitis | 0.19 |
| Erythema induratum | -0.08 |
| Lichen aureus | -0.08 |
| Hailey-hailey disease | -0.08 |
| Papuloerythroderma of ofuji | -0.08 |
| Tufted angioma | -0.08 |
| Tinea corporis | -0.08 |
| Cutaneous TB | -0.08 |
| Darier disease | -0.08 |
| Keratoacanthoma | -0.08 |
| Lichen striatus | -0.08 |
| Lichen simplex chronicus | -0.08 |
| Lichen planus | -0.08 |
| Sneddon Wilkinson disease | -0.08 |
| Melanoma | -0.08 |
| Congenital nevus | -0.08 |
| Pityriasis lichenoids et varioliformis acuta | -0.08 |
| Mycetoma | -0.08 |
| Filiariasis | -0.08 |
| Stasis dermatitis | -0.1 |
| Epidermoid cyst | -0.21 |
| Proliferating trichilemmal cyst | -0.21 |
| Nummular eczema | -0.21 |
| Atopic dermatitis | -0.21 |
| Scrotal calcinosis | -0.21 |
| Lichen nitidus | -0.21 |
